# A Short-term Comparison Between Result of Palisade Cartilage Tympanoplasty and Temporalis Fascia Technique

**Published:** 2012

**Authors:** Mahmood Shishegar, Abolhasan Faramarzi, Ayeh Taraghi

**Affiliations:** 1 *Department of otorhinolaryngology, Khalili Hospital, Shiraz University of Medical Sciences, Shiraz, Iran*

**Keywords:** Cartilage, Palisade, Temporalis fascia, Tympanoplasty, Tympanic membrane perforation

## Abstract

**Introduction:**

The use of cartilage as a grafting material has been advocated in cases where there is a high risk of graft failure, such as subtotal perforations, adhesive processes, and residual defects after primary tympanoplasties. The purpose of this study was to compare the graft acceptance rates and auditory outcomes of cartilage tympanoplasty operations using a palisade technique with those of primary tympanoplasty using temporalis fascia in a homogenous group of patients. Study Design: Prospective study.

**Materials and Methods:**

The study population included 54 patients who were operated on in two groups (palisade technique & temporalis fascia technique) with each group containing 27 patients. Patients with pure subtotal perforations (perforation of >50% of the whole tympanic membrane [TM] area), an intact ossicular chain, at least a one month dry period, and normal middle ear mucosa were included in the study. Grafts acceptance rates and pre- and post-operative audiograms were compared. The follow-up time was six months.

**Results:**

Graft acceptance was achieved in all patients (100%) in the palisade cartilage tympanoplasty group and in 25 patients (92.5%) in the temporalis fascia group. This difference was not statistically significant (P= 0.15). Comparison of the increases in mean speech reception threshold, air–bone gap, and pure-tone average scores between both techniques showed no significant changes.

**Conclusion:**

Our experience with the palisade cartilage technique demonstrates that subtotal or total perforation at high risk for graft failure can be treated efficiently, and that a durable and resistant reconstruction of the TM with reasonable auditory function can be achieved.

## Introduction

 Tympanoplasty is a procedure used to eradicate disease in the middle ear and to reconstruct the hearing mechanism (1). The principal aims of a tympanoplasty operation are to create an intact tympanic membrane (TM) and to restore functional hearing. Tympanoplasty techniques for chronic otitis media were first developed in Germany and the temporalis fascia was first used by Heermann (2). However, retraction or perforation after reconstruction of the eardrum is a well-known problem in middle ear surgery as the temporalis fascia can change its shape because of uneven shrinking and thickening, even on the fifth day following grafting (3). The instability of the temporalis fascia is critical in cases where perforations of the TM are large (4).

 The use of cartilage in the middle ear has been suggested for use on a limited basis to manage retraction pockets for many years (5). The array of different techniques developed, such as the perichondrial cartilage island technique, the palisade cartilage technique, the shield technique, the butterfly technique, and the crown cork technique, indicate the variety of methods used to surgically prepare the cartilage. It has been shown that large pieces of cartilage may twist after some years, so small palisades of cartilage are used (6). The palisade cartilage technique was first described by Heermann in 1962. The palisade technique has become popular in Europe, especially in Germany, and was proposed as the method of choice for recurrent defects of the TM (7). Cartilage is very useful for managing eustachian tube dysfunction that may cause graft failures and retractions (8). Autologous cartilage obtained from the ear (tragus or cymba) may resist the negative pressure because of its rigidity and convexity. So this method, because of the rigidity and stability of the cartilage, may be a better choice than using temporalis fascia in resisting the anatomic deformations caused by infection and middle ear effusion. It has been shown that cartilage is well tolerated by the middle ear, and long-term survival is the norm. Fascia and perichondrium need a new vascular supply but cartilage is supplied by diffusion. Cartilage also seems to offer high resistance both to lack of vascularization and to infections (9). 

Use of the palisade cartilage technique has been indicated in cases of subtotal perforations, adhesive processes (retraction pockets, adhesions and atelectiasis), tympanosclerosis, thermal perforations, and residual defects after primary tympanoplasties. The palisade cartilage technique is also resistant to the extreme barometric changes that occur during diving (10). It has also been shown that a palisade cartilage tympanoplasty provides restoration of the same level of auditory function as a tympanoplasty using temporalis fascia (11). To date, many authors have applied composite grafts of perichondrium cartilage and found no impairment of sound conduction in the ear (8). The aim of this article was to compare the graft acceptance rates and auditory outcomes of cartilage tympanoplasty operations using the palisade technique with those of primary tympanoplasties using temporalis fascia in two groups of patients.

## Materials and Methods

 The study population included 54 patients who were not selected according to age or sex. In all patients a unilateral subtotal TM perforation was detected. A total of 27 patients underwent a tympanoplasty using temporalis fascia, while in the other 27 patients palisade cartilage was used as a graft material to close the TM perforation. The indication for surgery was the presence of a unilateral pure subtotal perforation (perforation of >50% of the whole TM area), an intact ossicular chain, at least a one month dry period, and normal middle ear mucosa. Patients who had concomitant ossiculoplasty or any history of previous ear surgery were excluded from this study. 

**Fig 1:**
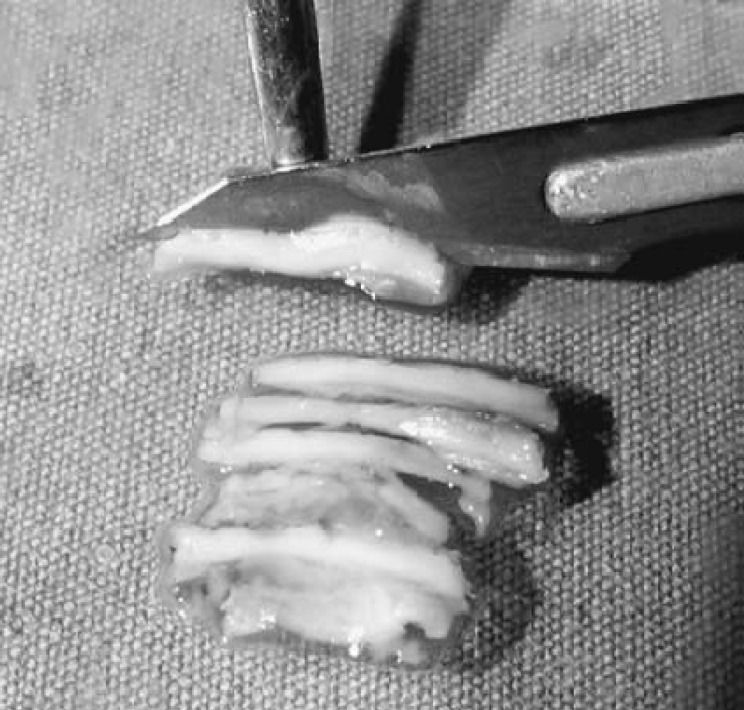
Photograph showing preparation of the cartilage

**Fig 2:**
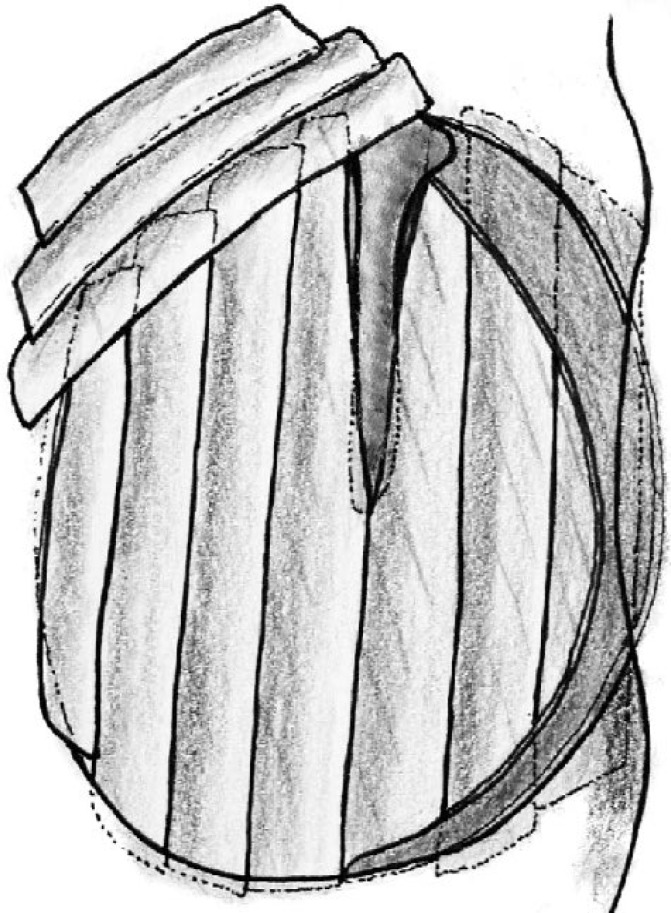
Schematic representation of the Palisade cartilage tympanoplasty

In the patients who underwent palisade cartilage tympanoplasty, conchal cartilage was used in all cases. The perichondrium was removed from one side of the cartilage, and the cartilage was then cut into several slices with, on average, four or five palisades placed in an over-under fashion (two placed anterior to the malleus handle and two or three placed posteriorly). The remaining perichondrium was left attached to the cartilage slices on the lateral side. The perichondrium layer removed at the beginning of the procedure was then laid on the cartilage palisades, so that all the unwanted small openings between the slices were covered to improve the healing process. In the patients who underwent tympanoplasty where the temporalis muscle fascia was used as a grafting material, the graft was harvested from the ipsilateral deep temporal muscle fascia and placed lateral to (over) the long process of the malleus and medial to (under) the drum remnant and anterior annulus. Gelfoam was placed both medial (to the middle ear) and lateral to the graft, and the wound was closed using absorbable sutures. Figure 1 shows the preparation of the cartilage strips and Figure 2 shows a schematic image of the palisade cartilage tympanoplasty.

Postoperatively, the patients were evaluated in a regular clinical manner and audiometrically at a six-month follow-up appointment. A successful tympanoplasty was defined as full acceptance of the graft, and intact healing of the TM without perforation, retraction, or lateralization within a follow-up period of six months from the operation. Auditory outcomes were evaluated using an audiogram. Audiological data were gathered from the preoperative and postoperative audiograms of the patients. The patients’ data were reviewed for changes in the pre- and postoperative air–bone gaps (ABG), which was defined as the difference between the preoperative and postoperative air–bone gap; pure-tone averages (PTA) at 500, 1000, 2000, 4000, and 8000 Hz; speech reception thresholds (SRT); and speech discrimination scores (SDS). Data analysis was performed using SPSS for Windows version 16 and the chi-squared test, Fisher’s exact test, and Student’s t-test for independent samples and paired samples were used for statistical comparisons. A *P* value of less than 0.05 was considered statistically significant. 

## Results

The patients’ ages ranged from 10 to 50 years with a mean of 30 years; 31 patients (57.4%) were female and 23 (42.6%) were male. In the patients who underwent palisade cartilage tympanoplasty 18 (66.7%) were female and 9 (33.3%) were male, and in the group who underwent fascia tympanoplasty 13 (48.1%) were female and 14 (51.9%) were male. In all patients a pure tone audiogram from 250 Hz to 8 KHz was obtained preoperatively. The follow-up period was six months postoperatively. Graft acceptance was achieved in all patients (100%) who underwent palisade cartilage tympanoplasty, whereas it was achieved in 25 patients (92.5%) in the temporalis fascia tympanoplasty group. This difference was not statistically significant according to the chi-squared test (P= 0.15). No graft failures were observed in the patients who underwent palisade cartilage tympanoplasty, but two graft failures were observed in the temporalis fascia tympanoplasty group. In addition, all postoperative perforations occurred in the patients who underwent temporalis fascia tympanoplasty. In both graft failures a small perforation developed at the central part of the TM but the cartilage strips resisted well resulting in an intact TM. There were no significant complications such as graft lateralization, blunting, or infection. 

The mean SRT change in the patients who underwent palisade cartilage tympanoplasty was not statistically different when compared to the functional gains in the temporalis fascia group (P=0.7). In each group the postoperative results were satisfactory. Also, a comparison of the mean ABG changes between the two groups was not statistically significant either (P>0.05). The audiometric results are shown in (Tables 1 to 3). Overall, a comparison of all the audiologic results between the two groups did not reveal any statistically significant differences.

**Table 1:** Audiometric results from patients who underwent temporalis fascia tympanoplasty

	Pre-operative (mean ± SD)	Post-operative (mean ± SD)	*P* value
SRT	30.0 ± 9.9	18.5 ±9.9	<0.001
SDS	94.6 ± 6.6	95.0 ± 7.8	0.5
ABG 250	33.5 ± 8.5	17.4 ± 7.4	<0.001
ABG 500	22.8 ± 9.8	10.6 ± 4.9	<0.001
ABG 1000	18.0 ± 7.8	9.6 ± 3.4	<0.001
ABG 2000	18.1 ± 6.1	8.1 ± 3.4	<0.001
ABG 4000	27.6 ± 9.2	13.5 ± 6.8	<0.001
ABG 8000	32.4 ± 15.4	25.0 ± 17.5	<0.001

ABG, air–bone gap; SDS, speech discrimination score; SRT, speech reception threshold

**Table 2:** Audiometric results from patients who underwent palisade cartilage tympanoplasty

	Pre-operative (mean ± SD)	Post-operative (mean ± SD)	*P* value
SRT	34.1 ±10.0	19.4 ± 7.8	<0.001
SDS	94.1 ± 8.3	96.7 ± 6.5	0.03
ABG 250	37.5 ± 10.5	16.8 ± 6.7	0.001
ABG 500	30.1 ± 13.9	11.1 ± 6.4	<0.001
ABG 1000	23.1 ± 6.8	11.3 ± 3.8	<0.001
ABG 2000	18.8 ± 10.5	7.2 ± 5.1	<0.001
ABG 4000	29.1 ± 5.0	17.0 ± 7.2	<0.001
ABG 8000	32.6 ± 10.1	25.9 ± 10.7	<0.001

ABG, air–bone gap; SDS, speech discrimination score; SRT, speech reception thresho

**Table 3:** Comparison of post-operative audiometric results between the two patient groups

	Operation	Mean ± SD	*P*
SRT	Palisade Fascia	19.4 ± 7.8 18.5 ± 10.0	0.7
			
SDS	Palisade Fascia	96.7 ± 6.6 95 ± 7.8	0.3
			
ABG 250	Palisade Fascia	16.8 ± 6.7 17.4 ± 7.4	0.7
			
ABG 500	Palisade Fascia	11.1 ± 6.4 10.5 ± 4.9	0.7
			
ABG 1000	Palisade Fascia	11.2 ± 3.8 9.6 ± 3.4	0.09
			
ABG 2000	Palisade Fascia	7.2 ± 5.1 8.1 ± 3.4	0.4
			
ABG 4000	Palisade Fascia	17.0 ± 7.2 13.5 ± 6.8	0.07
			
ABG 8000	Palisade Fascia	25.9 ± 10.7 25 ± 17.5	0.8

ABG, air–bone gap; SDS, speech discrimination score; SRT, speech reception threshold

## Discussion

The use of cartilage is experiencing a renaissance in ear surgery because it appears to offer an extremely reliable method for reconstruction of the TM in cases of advanced middle ear pathology and eustachian tube dysfunction. In this short-term study patients with subtotal perforations (perforation of >50% of the whole TM area), an intact ossicular chain, at least a one month dry period, and normal middle ear mucosa were included. The graft acceptance rate was 100% for the patients who underwent a palisade cartilage tympanoplasty and 92.5% for the patients who underwent fascia tympanoplasty; this difference was not statistically significant. Our results are comparable to other studies. For example, Neumann and colleagues reviewed 84 cases of patients who underwent palisade tympanoplasty, with mixed pathologies such as cholesteatoma, adhesive processes, subtotal perforations, and chronic mesotympanal otitis, and found an overall graft acceptance rate of 97.6% (12). Uzun and colleagues achieved 100% (0/14 perforations) TM closure with type 1 palisade cartilage grafting, whereas a 84.2% (3/19 perforations) success rate was observed in type 1 tympanoplasties with temporalis fascia grafting in children aged 5 to 15 years with tensa cholesteatoma (13). Anderson and colleagues compared the results of fascia and palisade cartilage grafting after surgery for either tensa or sinus retraction cholesteatoma in children. No perforations were found in patients following palisade cartilage tympanoplasty, whereas there were four perforations in the patients who underwent fascia tympanoplasty (11). 

In our study, auditory function in palisade cartilage tympanoplasty patients was not statistically different when compared to the gains observed in the patients who underwent temporalis fascia tympanoplasty. Other studies in the literature have also reported good or acceptable hearing results with cartilage grafting. Cagdas Kazikdas and colleagues demonstrated that a comparison of the gains in mean speech reception threshold, air–bone gap, and pure-tone average scores between the palisade cartilage and fascia technique showed no significant differences (14). Following cartilage-perichondrial composite graft tympanoplasty Levinson reported that 65% of his patients had closure of the ABG to within 10 dB and 86% to within 20 dB (15). In a study by Dornhoff, no significant differences were demonstrated in gains in auditory function in patients who had cartilage-perichondrium grafting compared with patients who had grafts of perichondrium alone (16). Kirazli and colleagues also found no significant difference between the audiologic results after cartilage perichondrium and temporalis fascia tympanoplasty (17). Similarly, a study by Cabra and colleagues observed no relevant differences between the functional results of the two procedures (palisade cartilage and fascia tympanoplasty) (18). 

Zahnert and colleagues concluded that the ideal acoustic thickness of cartilage should be approximately 0.5 mm (19). The full thickness is 0.7 to 1 mm. However, thinning the cartilage makes the reconstruction process more difficult due to the inevitable twisting of the cartilage. We applied full thickness cartilage in our procedure. In a similar study, Ozbek and colleagues used full-thickness strips of tragal cartilage in palisade tympanoplasty in the children, which resulted in good auditory outcomes in the cartilage tympanoplasty patients that were comparable to those in the fascia group (20). Experimental histopathologic studies have shown that cartilage is stable because of the fibrile structure of the matrix, which is independent of the survival of cellular elements (21,22). 

Reconstruction of the TM using the palisade cartilage technique in tympanoplasties allowed us to achieve good anatomic and audiologic results that were at least similar, if not better than, traditional methods of reconstruction in high-risk cases.

## Conclusion

The results of this study are in favor of using the palisade cartliage technique in difficult cases. The outcomes in our patient series indicate that cartilage tympanoplasty achieves good results. Cartilage a very effective material for the reconstruction of the TM and grafts can provide an excellent anatomical result, perfect stability, and good functional outcomes. 
